# A review of thanatosis (death feigning) as an anti-predator behaviour

**DOI:** 10.1007/s00265-017-2436-8

**Published:** 2018-01-15

**Authors:** Rosalind K. Humphreys, Graeme D. Ruxton

**Affiliations:** School of Biology, University of St Andrews, Dyer’s Brae House, St Andrews, Fife, KY16 9TH UK

**Keywords:** Thanatosis, Tonic immobility, Death feigning, Anti-predatory defence, Trade-offs

## Abstract

**Abstract:**

Thanatosis—also known as death-feigning and, we argue more appropriately, tonic immobility (TI)—is an under-reported but fascinating anti-predator strategy adopted by diverse prey late on in the predation sequence, and frequently following physical contact by the predator. TI is thought to inhibit further attack by predators and reduce the perceived need of the predator to subdue prey further. The behaviour is probably present in more taxa than is currently described, but even within well-studied groups the precise taxonomic distribution is unclear for a number of practical and ethical reasons. Here we synthesise the key studies investigating the form, function, evolutionary and ecological costs and benefits of TI. This review also considers the potential evolutionary influence of certain predator types in the development of the strategy in prey, and the other non-defensive contexts in which TI has been suggested to occur. We believe that there is a need for TI to be better appreciated in the scientific literature and outline potentially profitable avenues for investigation. Future use of technology in the wild should yield useful developments for this field of study.

**Significance statement:**

Anti-predatory defences are crucial to many aspects of behavioural ecology. Thanatosis (often called death-feigning) has long been an under-appreciated defence, despite being taxonomically and ecologically widespread. We begin by providing much-needed clarification on both terminology and definition. We demonstrate how apparently disparate observations in the recent literature can be synthesised through placing the behaviour within a cost-benefit framework in comparison to alternative behavioural choices, and how aspects of the ecology differentially affect costs and benefits. Extending this, we provide novel insights into why the evolution of thanatosis can be understood in terms of coevolution between predators and prey. We offer further novel hypotheses, and discuss how these can be tested, focussing on how emerging technologies can be of great use in developing our understanding of thanatosis in free-living animals.

## Introduction

Predation is fundamental to the lives of wild animals, influencing key aspects of fitness such as feeding, breeding, and often, ultimately, mortality. In response to the threat of predation, and the fitness costs to an individual associated with such predation, natural selection has resulted in the evolution of a wide variety of morphological, physiological, chemical and behavioural defensive adaptations in prey species. Very few animals are immune to substantial threat of predation in at least one stage of their ontogeny. As such, the many and sometimes complicated adaptations involved in predator-prey interactions are of great interest to behavioural ecologists. An interaction between a predator and prey individual can often usefully be broken down into a sequence of stages, beginning with the two individuals being in proximity, and leading through detection, identification, reducing separation, contacting, subduing and finally consuming (Endler 1991; Caro 2005). There is a diversity of mechanisms that prey have evolved to avoid attack by predators, some of which require deployment at particular stages in the attack sequence in order to be effective. One interesting and well-known defence that prey can exhibit late in the sequence of a predation event is tonic immobility (hereafter shorted to TI). TI has been discussed in the scientific literature for over a century (e.g. Fabre 1900); but until recently most studies were observational and qualitative (see Edmunds 1974 for an insightful synthesis of these earlier works and Ruxton et al. 2004). As a defensive mechanism, TI can be viewed as a ‘last resort’, occurring not only after the prey has already been detected by the predator but most often following physical contact between the predator and its prey. This makes it quite distinct from immobility used to reduce the risk of predator detection or tracking, since such “freezing” occurs much earlier in the sequence of a predatory attack. That is, we consider stillness that enhances the effectiveness of camouflage or masquerade to be a different process from TI. Further some animals (e.g. pill bugs, armadillos) adopt a curled posture that makes vulnerable body parts inaccessible to predators. We consider this, too, a separate process to TI, which does not rely on making the prey physically less vulnerable to the predator.

TI typically occurs when animals are physically restrained, and it involves prey adopting a relatively immobile state that can last—from seconds to hours—even after the physical constraint has been released. In some vertebrates, the behaviour can involve reduced breathing rates, bradycardia, tongue protrusion, and setting the eyes wide open—features very reminiscent of dead individuals of that species. TI is, therefore, also known as death feigning, playing dead, playing possum, animal hypnosis, and thanatosis. By offering the following definition of TI, we hope to clarify the scope of our current study:

*Tonic Immobility (TI) is the unlearned adoption of a motionless posture by a prey individual triggered by physical contact or very close proximity of – not injury inflicted by – a predator (or other antagonist). The posture does not reduce the sensory ability of the predator to locate or identify the prey, or reduce the physical vulnerability of the prey if the attack is pursued. The state of motor inhibition is maintained for a time even after release by the predator, and when in this state the prey exhibits reduced responsiveness to external stimulation (although monitoring of the environment can still occur). In the absence of mortality or injury during TI, the prey can recover its original physiological state on emerging from TI.*

Notice that this definition is agnostic as to the function of TI. Although it is generally assumed that TI confers an anti-predatory benefit, in causing the predator to break off its attack, we feel this function is not necessary for a clear and effective definition. Indeed, our definition makes no assumption about predatory response to TI. Nor does it make any assumption about the underlying drivers of any predatory response. Our definition is also agnostic as to whether the predator “believes” or otherwise acts as if the prey is dead. It is for this reason also that we prefer to refer to the behaviour as tonic immobility (TI) rather than thanatosis (from the Greek noun θανάτωσις meaning “putting to death”) or death feigning.

As to function, it is generally assumed that after any active resistance to attack has failed, and with the possibility of fleeing removed, TI can serve to inhibit further attack by predators and reduce the perceived need of the predator to subdue prey further. There is, as with most biological mechanisms, great variation across species and between individuals in the nature of TI exhibited. As an example, physical contact is not always essential to trigger the behaviour—various birds have been reported as going into an immobile state when they sense an imminent threat of attack without an opportunity for escape (Vogel 1950). The phenomenon is widespread taxonomically, but is one of the more difficult topics for study in the field of predator-prey interactions (particularly in vertebrates). Because it is manifest very late in the predation-sequence, there are ethical challenges associated with its study in the laboratory and logistical challenges in the field, where naturally-occurring prey captures are spatio-temporally unpredictable and often inhibited by proximity of human observers.

Despite being discussed as far back as the nineteenth century, notably in a posthumously published essay of Darwin’s (1885), TI has not yet been the subject of intensive study. There were some papers by researchers from Germany on the subject from early in the twentieth Century (e.g. Bleich 1928), but TI remained a more obscure and rather side-lined anti-predatory behaviour throughout most of the 1900s. However, in the past 20 years there has been a surge of more purpose-designed study of this phenomenon, and so we believe that a review of this intriguing defence is timely. Here we aim to review the latest and most important research on TI, as we consider its distribution, form, functions, trade-offs and evolution. Where we can, we highlight what we see as being the most interesting and useful areas for future work.

## Taxonomic distribution

TI has been described—often anecdotally—in a wide range of taxa, but there remain relatively few papers presenting quantitative accounts of the phenomenon. In the invertebrates, it has been suggested to occur (at least) in: crustaceans, stick insects, spiders, butterflies, stoneflies, water-scorpions, cicadas, crickets, mites, beetles, damselfly larvae, ants, bees and wasps (see Cassill et al. 2008 for a partial list and references, and the following for a few more recent invertebrate examples: Coutinho et al. 2013; Ritter et al. 2016; Cadena-Castañeda et al. 2016; Neves and Pie 2017). In the vertebrates, it has been recorded in mammals, birds, reptiles, amphibians and fish (again, see Cassill et al. 2008 for a partial list and references, and the following for a few more recent vertebrate examples: Gally et al. 2012; Marques et al. 2013; Sannolo et al. 2014; Batista et al. 2015; Muscat et al. 2016; Sanchéz Paniagua and Abarca 2016; Patel et al. 2016; de Castro et al. 2017; Freret-Meurer et al. 2017). Even within well-studied vertebrate groups, though, the precise distribution of the behaviour is unclear—there are probably a few reasons for this.

Firstly, the lack of associated specialist morphological adaptations means that any observer must be present at the moment the behaviour occurs in order to have confidence in its presence in an individual’s, or species’, defensive repertoire. Even though TI has become co-opted into larger defence strategies in some species, such as aposematic warning or camouflage colouration (Rogers and Simpson 2014), there is no evidence of a strong correlation between TI and traits associated with these other defensive strategies. Secondly, as a last line of defence for prey species (usually occurring after the predator has made physical contact with the prey) its observation is logistically challenging in the field, due to the temporal and spatial unpredictability of natural predation. Additionally, human presence often serves to deter natural predators, hindering the likelihood of success of field studies further. Adding to this problem, staged encounters involving vertebrates in laboratory or otherwise man-made study environments replace—to some extent—practical challenges with ethical challenges of their own. Finally, the use of TI as a defence often seems highly variable, even within a local population, and very patchily distributed at higher levels of organisation. Failure to observe the behaviour in most species of a given family therefore would not automatically inspire confidence in its likely absence from the remaining species. Likewise, its apparent prevalence in most species of a given family would not mean that it could be assumed to be present in all species of that family. As an example of this, TI seems relatively prevalent amongst snakes but the number of families involved remains unclear (Gregory et al. 2007).

Speculating about the underlying patterns of TI’s taxonomic distribution is, therefore, made very difficult by the sparse reports of the behaviour. For some taxa, it is tempting to speculate about trends of prevalence. As an example, perhaps TI might be more prevalent in snakes because they are generally not especially fleet of movement relative to mammalian and avian predators (reducing the efficacy of fleeing as an alternate anti-predator strategy), and their elongate bodies may facilitate contact by a predator. However, speculations such as this remain no more than “just so” stories at this stage. We can suggest, though, that because TI requires no anatomical specialisations there seems to be little cost to carrying the potential to use this last-ditch defence when under attack. Any costs associated with implementing TI will only be paid in circumstances when a predator has completed almost all of its predatory sequence on a prey individual, and often when the prey is actually in the clutches of the predator. As such, we might expect such costs to be paid relatively infrequently. Further, the likelihood of prey displaying TI behaviours may be more closely linked to traits of the predator than the prey. In particular, as will be discussed further in section 6 ‘Coevolutionary considerations’, we think it worthy of further study that TI may be more commonly implemented against generalist predators with a broad diet, and predators which encounter prey simultaneously or in quick succession (or which are otherwise constrained in how much time they can devote to ensuring that contacted prey are dead or permanently immobilised).

The fact that TI is mainly defined by a lack of behaviour and does not involve specialist anatomical adaptations also suggests that it is a relatively simple defence to evolve. Given this simplicity and the widespread but sparse nature of its taxonomic distribution, it seems probable that TI has evolved a large number of times. This seems more plausible than explanations based on one evolution and multiple losses of the trait. Given multiple independent evolutions, one might expect considerable variation in the form and usage of TI. There does seem to be strong evidence for such variation and this (together with the issues of almost certain under-reporting) makes generalisations challenging. However, there is evidence—discussed in later sections—that TI is partly under genetic control and is often consistent within an individual but variable within- and between-populations in the form and frequency of the behaviour.

Individual consistency of TI can often be linked to other traits. As an example, Krams et al. (2014) reported high repeatability in aspects of TI in the study of a laboratory-reared population of the mealworm *Tenebrio molitor*. They found that individuals with a higher metabolic rate took a longer time to enter a state of TI when threatened and tended to remain immobile for a shorter time. This complex of traits was repeatable within an individual, not only over time but also across environmental contexts. Similarly, Edelaar et al. (2012) found consistency of TI traits within an individual in a study where individuals of two avian species (yellow-crowned bishop *Euplectes afer* and tree sparrow *Passer montanus*) were exposed both to mounts of predatory birds placed near their home cage and to repeated handling. The duration of TI was here found to be consistent within an individual, with shorter durations correlated with higher activity rates in the presence of the mount. Edelaar et al. interpret these two traits—TI duration and activity rate—as being indicative of general individual “boldness”.

Considering interspecific differences in TI behaviours of animals with different histories of exposure to predation, Suzuki et al. (2013) found lower TI responses to physical restraint in Bengalese finches (*Lochura striata var. domestica*) compared to the white-backed munia (*L. striata*), from which they were domesticated some 250 years ago. Bengalese finches also had lower corticosterone levels. The authors interpreted these results as being suggestive that artificial selection during domestication has led to reduced fear responses; with less exposure to predators during domestication, prey will experience reduced selective pressures for such drastic last-ditch anti-predator defence strategies like TI. Evidence that TI is linked to a fear response also comes from the study of Fijian ground frogs (*Platymantis vitiana*) by Narayan et al. (2013). In this, frogs were exposed to a range of experimental manipulations. Those manipulations that experimenters expected to simulate the highest levels of predatory threat—such as sight of a predator—induced both higher levels of circulating corticosterone and stronger TI behaviours in response to standardised handling.

TI has also been found to vary in occurrence over ontogeny in some species. As a generality, those life-history classes less able to use fleeing or active resistance as defences are more likely to use TI. Thus, there is a tendency in some taxa at least for it to be associated with early life-history stages. In the fire ant *Solenopsis invicta*, for example, while day-old workers respond to encounters with workers from another nest by exhibiting TI, week-old workers instead respond with aggressive behaviours (Cassill et al. 2008). In this species, the still-soft exoskeleton of the day-old workers probably renders their ability to inflict damage on alien workers very low, but their vulnerability to damage very high. Hence, TI appears to be the more effective strategy for the earlier fire ant life stage and, indeed, day-old workers were four times more likely to survive such an encounter than week-old individuals encountering similar aliens (Cassill et al. 2008). The authors of this study speculated that TI is effective partly because aggression between workers from two different colonies often involves multiple individuals. Here there may be selection to attack as many alien workers as possible, which may come at the expense of failing to ensure that immobile individuals are actually dead. TI can therefore increase the likelihood of individual survival in such situations.

Interestingly, a recent study by Matsumura et al. (2017) using red flour beetles, *Tribolium castaneum* and *T. confusum*, demonstrated that a behavioural correlation between TI duration and walking distance can decouple across life stages. Walking distance here can be used as a measure of mobility and can go some way to indicate the likelihood of prey escaping attack by fleeing. They found a negative correlation of traits in adults of both the holometabolous species, but not in larvae of either species. The authors suggest that the negative correlation between TI and walking is decoupled across life stages, contrasting with the phenotypic correlations between behavioural traits that have been found to be maintained from larvae to adults in hemimetabolous insects. Metamorphosis is, therefore, suggested to have the potential to change trade-offs between behavioural traits, including the duration of TI.

Linkages with other life-history traits have also been observed across many different taxa. For example, Gregory and Gregory (2006) reported that gravid garter snakes (*Thamnophis elegans*) were much more likely to show TI than similar-sized non-gravid females. This may be due to the impaired locomotive ability and flexibility of gravid individuals making fleeing or resisting less effective as alternative tactics (Gregory and Gregory 2006). Other traits that have been shown in some species to influence TI include sex (Miyatake 2001a, b), size (Hozumi and Miyatake 2005; Farkas 2016), conditions during rearing (Tojo 1991), and sexual history (Kuriwada et al. 2009a, 2011). The effect of these traits on the occurrence and duration of TI behaviours can generally be interpreted in terms of shifting costs and benefits of the tactic; this will be discussed further in section 5 ‘Adaptive significance and cost-benefit analyses’. However, in keeping with an emerging theme in TI research, these life-history correlates can be explained in individual species or populations but are not seen universally across species. As an anti-predator defence, there is still clearly a lot to discover about the occurrence, patterns and evolution of TI.

## Form

### Physiological mechanisms

A number of different physiological mechanisms can be involved in controlling TI. In vertebrates, reduced breathing rates, bradycardia, salivation, defecation and urination are common during TI, and are consistent with mediation by the parasympathetic nervous system (Rogers and Simpson 2014). The role of serotonin can be complex, either attenuating or enhancing TI depending on species, dose and site of action. Additionally, it has been found that the stress hormone corticotropin-releasing factor (CRF) increases the duration of TI when injected into the brain’s amygdala, a region important for regulating emotional state (Rogers and Simpson 2014). Neurochemicals that affect motivational state have also been found to affect TI. For example, dopamine receptor antagonists increase TI duration, while dopamine agonists decrease TI duration, in both birds and mammals (Rogers and Simpson 2014).

In the laboratory, TI can be induced by handling and manually restricting an animal’s movements. This has led to some detailed observations in a range of vertebrate species, including mammals, fish, and birds. As a mammalian example, Donatti and Leite-Panissi (2011) studied the role of CRF system in control of emotional responses in guinea pigs (*Cavia porcellus*) through microinjections into the central amygdaloid (CeA) and basolateral amygdaloid (BLA) nuclei. The use of CRF, which serves as an agonist of CRF receptors, increased the duration of TI while inhibition of the CRF receptors, by microinjection of alpha-helical-CRF_9_–_41_, decreased the duration of TI. The activation of CRF receptors of the BLA or the CeA are, therefore, key in the modulation of innate fear, increasing TI duration in guinea pigs (Donatti and Leite-Panissi 2011).

Some sharks’ TI behaviours have also been induced and described through restraining manipulations (Whitman et al. 1986; Watsky and Gruber 1990; Davie et al. 1993; Henningsen 1994). In investigating the stress physiology of extended duration TI in the lemon shark (*Negaprion brevirostris*), Brooks et al. (2011) reported that the stress of experiencing TI impacted a number of blood chemistry parameters and reduced breathing efficiency. However, the authors also found that a series of compensatory mechanisms appear to counter these effects. In response to the reduced short-term ventilation efficiency induced by TI, there was a significant increase in ventilation rates compared to in control animals, increasing the capacity for gas exchange. Additionally, Brooks et al. postulate that physical adaptations to the gills and circulatory system, associated with stress responses, were sustained during TI. These adaptations include the recruitment of additional gill lamellae and vasodilatation of branchial blood vessels, and would serve to further facilitate the decline of carbon dioxide concentrations.

The physiology of TI has also been relatively well-studied in birds, perhaps most prolifically in domestic fowl (*Gallus gallus domesticus*) (see Jones 1986). Recently, a study with pigeons (*Columba livia*) has begun to uncover the mesencephalic circuitry involved in the control of TI in birds. Melleu et al. (2017) report the consequences of TI induction on the distribution and density of c-Fos labelling (which serves as a marker for neural activity) in specific regions of pigeons’ brains. They found that the induction of TI responses, by restraining birds, increased c-Fos expression in the ICo/GCt of the pigeon’s brain complex, suggesting that ICo neurons may be important for the expression of TI. Interestingly, Melleu et al. suggest that the functional similarity between the bird and mammalian mesencephalic circuits mediating fear and defensive behaviours may mean that they are substantially conserved in amniote species, as a response to inescapable threatening stimuli.

In insects it is typical that ventilatory movements of the abdomen strongly decrease during TI, while slow flexor and extensor motor neurons fire tonically to maintain stiff posture (see Rogers and Simpson 2014). As an example, these responses both occur in crickets (*Gryllus bimaculatus*), however heart rate during TI in this taxa appears to be double that of the resting state (Nishino and Sakai 1996; Nishino 2004). Rogers and Simpson (2014) describe the control of TI in crickets, separating the process into two distinct phases: induction and maintenance. Physical restraint induces TI, leading to the production of vigorous resistance reflexes driven by bursting activity by excitatory motor neurons. In turn, this produces a build-up of isometric tension in the flexor muscles, triggering the maintenance phase. The maintenance phase is characterised by the rapid cessation of fast motor neuron activity, leaving only slow flexor motor neurons active (see Rogers and Simpson 2014).

In other invertebrates, such as *Tribolium* beetles and the spider *Larinoides cornutus*, neurochemicals like dopamine and octopamine have been found to be key influencers of TI duration (see Rogers and Simpson 2014). The effect of biogenic amines on TI has been well studied in *Tribolium* beetles in particular. In the confused flour beetle (*Tribolium confusum*) and the red flour beetle (*T. castaneum*) brain dopamine levels are lower in strains exhibiting higher frequency and longer duration TI (L-strain) than strains exhibiting lower TI frequencies and shorter TI durations (S-strain) (Miyatake et al. 2008a; Nakayama et al. 2012). Additionally, caffeine administration has been found to decrease TI duration in L-strain *T. confusum* and *T. castaneum* and, since caffeine is known to activate dopamine, the duration of tonic immobility is very likely affected by dopamine via dopamine receptors in these species (Nishi et al. 2010; Nakayama et al. 2012). As more taxa are studied in the future for their TI behaviours, both vertebrates and invertebrates, we expect that even more physiological mechanisms associated with the anti-predator strategy will be uncovered.

### Mechanisms involved in conferring protection

There are a number of mechanisms by which TI might confer protection from predators; these are enumerated by Miyatake et al. (2009) and are potentially complimentary. One obvious potential mechanism is that the immobile state might actually be death feigning, where the predator has an aversion to long-dead prey (which might be adaptive because of risk from toxins produced by microbial spoilage). In this case, TI causes the predator to mistakenly reject a live individual because it is misidentified as a long-dead individual. An implication of this is that the predator must have very simple cognitive functioning in this regard, since the individual that now appears dead was obviously very much alive moments earlier.

Miyatake et al. (2009) argue that another mechanism might be that TI simply reduces the predator’s ability to localise the prey individual, relative to a moving individual. This may be at least part of the explanation in cases where prey first drop from a plant to the ground (or otherwise physically distance themselves from a potential predator) ahead of inducing a state of TI. Dropping may hide a prey individual in an underlying, more camouflaging substrate—such as dead leaves or mud—and subsequent stillness would add to the camouflaging effect. However, as discussed previously, we think it is useful to differentiate TI from camouflage or masquerade.

Some authors have suggested that TI may serve as a physical defence against predators that swallow prey whole. For example, this might work by TI involving assuming a posture that makes swallowing more difficult or impossible. Honma et al. (2006) report this occurring in their staged interactions between the pygmy grasshopper *Criotettix japonicus* and the frog *Rana nigromaculata*. When faced with the gape-limited frog predator, the grasshopper adopted a pose that effectively increased its functional body size and exposed its spines. These adaptations made the grasshopper impossible to swallow for smaller frogs that could manage to swallow it when it was adopting its normal posture. For slightly larger frogs, ultimate swallowing was only possible after time-consuming manipulation to position the immobilised prey correctly in the mouth, hence making eating this prey less attractive from a foraging rate efficiency perspective. In this regard, it is significant that a field-based aspect of Honma et al.’s study found that larger frogs that could consume the grasshoppers undergoing TI in the laboratory environment did not consume them in a field situation where other grasshopper prey were available in abundance. In another interesting aspect, the grasshoppers never showed this behaviour in response to bird, spider or mantid predators that do not need to consume their prey whole; this behaviour therefore appears to have developed as a specialised response to a particular type of predator. In an earlier study, Moore and Williams (1990) observed stonefly (*Pteronarcys dorsata*) nymphs curl up into a ball in a way that projected spines and prevented ingestion by fish predators. Curling into a ball can serve to shelter vulnerable body parts from exposure to a predator. Examples of where predators are presented only with an armoured shell are widely known, from insects such as pill-bugs all the way to large vertebrates like the armadillos. However, as we argued previously, we think it useful to separate deployment of physical defences that render handling the prey more difficult or impossible for the predator as a separate process from TI. To us, TI acts on the cognitive systems of the predator, triggering a decision to attack less vigorously, but this decision by the predator is not linked to the prey making itself physically harder to handle.

It has also been proposed that TI could confer protection from predators through being combined with the production of post-ingestion aversive secretions, that trigger regurgitation, if the assumed posture minimises prey injury during the processes of ingestion and regurgitation. Toledo et al. (2010) provide supporting reports for this where frogs have survived regurgitation after having been swallowed whole by snakes; the immobile postures of the frogs appear to have protected them from more serious wounds and, following their production of noxious secretions inside the predator, they have been ejected and able to escape alive. However, again because the protection is essentially physical, we think it is best to differentiate this strategy from TI as we define it here.

As another possible underlying mechanism of TI, it may be that some predators have a relatively hard-wired sequence of actions that they need to perform during prey capture. In this case, TI might subvert this sequence of actions by denying the predator successful completion of a subjugation action and, thus, preventing them from initiating later actions of the predation sequence. Indeed, this may cause the predator to break off the attack entirely and abandon the interaction with that particular prey item. However, clearly such maladaptive behaviour from the predator’s perspective could only be sustained if the prey concerned were encountered infrequently, such that countermeasures had not evolved. Further, we know of no examples of this being demonstrated.

It could also be that TI sometimes functions as an aposematic signal to predators that the prey is chemically or otherwise well-defended, and thus unattractive as a food item. However, the one study to test this possibility found no evidence that this was the case (Miyatake et al. 2009).

A further potential mechanism by which TI may confer protection from predators occurs if moving prey are more salient to a predator’s senses; in these circumstances, then it may be that TI is a means of deflecting a predator’s attention to alternative prey in a situation where prey are exposed simultaneously to the predator. Strong evidence for this was presented by Miyatake et al. (2009), who also pointed out that this mechanism was not the only one acting in their study system, since TI also offered protection when prey were offered singly to the predator (this study is discussed further in section 4 ‘Evidence of anti-predatory function’).

More generally, TI might be effective in any circumstances where there is a cost to predators in time invested in making sure that a captured individual is definitely dead (or at least immobilised). The most obvious circumstance for this to occur is when a predator has potential to capture several prey items but only a short period of time when those prey are available; for example, when a predator discovers a group of prey that take some time to flee to cover. Time spent making sure that one prey item has been immobilised may have an opportunity cost in reduced likelihood of obtaining other prey. Hence, we speculate that it may sometimes be more beneficial to a predator to abandon a prey item that appears immobile or dead in order to take down other prey items while they are abundant before pausing its hunting session to check on its bounty and feed.

There are clearly multiple, and probably complimentary, mechanisms through which TI could inhibit further attack by a predator and, thus, increase the likelihood of prey survival. In the next section we will move on to discuss some evidence for TI’s anti-predatory function.

## Evidence of anti-predatory function

There is strong evidence that suggests insects utilise TI as a defence against predators. In a study of a laboratory population of yellow mealworm beetles (*Tenebrio molitor*), for example, Krams et al. (2013a) found that resting metabolic rate (RMR) was consistent within individuals. High metabolic rate was correlated with both longer latency to show TI in response to experimental jarring with the substrate (caused by experimenters striking the experimental arena) and reduced duration of TI. After RMR and TI were measured, beetles were moved to another arena into which an insectivorous bird was introduced. High-RMR individuals were preferentially consumed by the bird. The authors interpret these results in terms of high-RMR being part of a “bold” personality type that will experience increased predation risk (because they exploit the TI strategy less strongly) but obtain benefits in terms of higher activity and thus increasing mating opportunity. A subsequent study observed essentially similar results with a nocturnal predator: the brown rat (*Ratus norvegicus*) (Krams et al., 2013b). For both predators, the beetles could probably use vibrations through the substrate to warn of danger of predation. In another example, Miyatake et al. (2009) and Nakayama and Miyatake (2010a) bred lines of the red flour beetle *T. casteraneum* for either longer duration and higher frequency TI (the L-strain) or shorter and less frequent (the S-strain), and demonstrated that L-strain individuals suffered less from predation in an experimental arena with a Adanson’s house jumping spider (*Hasarius adansoni*). Further evidence of TI offering insects protection from predators is discussed in later sections.

There is currently less evidence for TI’s anti-predatory function in vertebrates as studies involving predators and vertebrate prey are understandably rarer for ethical reasons. Our reporting of the results of the following two studies should in no way be seen as endorsement of these experiments from an ethical standpoint. Our consideration of them most certainly should not be seen as in any way encouraging any further experiments involving staged encounters between vertebrate predators and prey to further our understanding of TI. The first study we will mention was reported by Thompson et al. in 1981, where a domestic cat (*Felis domesticus*) was introduced into an arena containing two Japanese quail (*Coturix coturix japonica*). One quail had been induced into a state of TI by constraint by a human, the other was unrestrained and mobile. Four different cats were used, each being used in four trials. In 14 of 16 trials the first bird attacked by the cat was the mobile individual. In a further experiment, a cat was introduced into an arena containing two mobile, unconstrained quail (again, each of the four cats was used four times, each with two fresh quail). The cat was allowed to attack the quail, attacks by the cat were not always fatal, and TI was induced by these predatory attacks on 18 occasions. The authors described the response of the predator to TI adoption in a prey item:

“After TI was induced in a bird, the cat would leave it to stalk the remaining moving bird. A bird would be re-attacked if it came out of TI during a trial. In one case, a bird was re-attacked, killed and partially eaten after it came out of TI and moved while the second bird was still in TI.”

Another study utilising birds as vertebrate prey species in investigating TI is that of Sargeant and Eberhardt (1975). They exposed 50 ducks (of 5 different species, some pen-reared and some wild-caught) singly or in small groups to predation by captive red foxes (*Vulpes fulva*) and concluded that TI by ducks was an effective anti-predatory tactic. They observed that: most ducks survived their first encounter with a fox, the majority survived several attacks, and that TI was the near-ubiquitous response to physical contact by a fox. However, this study lacked a systematic exploration of whether availability of alternative prey influenced the responses of foxes to ducks exhibiting TI. The uniformity of behaviour reported does not allow us insight into how foxes would have reacted to a duck that did not show TI. There is a clear need for further studies into vertebrates’ use of TI as an antipredator defence and, as discussed in section 8 ‘Future challenges and opportunities for research’, new technologies—such as on-board cameras—may prove incredibly useful in developing our understanding of wild and natural predator-prey interactions.

As mentioned above, our discussion of these experiments involving staged encounters between vertebrate predators and prey in no way endorses these kinds of studies from an ethical standpoint. Technological advances in on-board cameras and telemetry have the potential to allow the study of TI to be advanced by means of observation of naturally-occurring predator-prey encounters—but even such studies should be approached very carefully from an ethical standpoint.

## Adaptive significance and cost-benefit analyses

Despite the potential anti-predator benefits that could be associated with adopting TI behaviours (discussed above), there are also likely to be related costs. Some of these costs will impact evolutionary trade-offs in species, influencing variation in TI expression and its prevalence. Alternative last-ditch anti-predator behaviours, such as struggling to break free and fleeing or aggressively attacking the would-be predator, will also probably have benefits and costs that will influence their expression. Hence, we might expect the prevalence and strength of TI to reflect conditions where the cost-benefit trade-off favours TI over alternative behavioural tactics. As an example of this, Gyssels and Stoks (2005) found that TI in larvae of the damselfly *Ischnura elegans* was more likely to be utilised in staged laboratory encounters with predators if an alternate anti-predator defence was negated by removal of lamellae; otherwise, lamellae can be sacrificed through autotomy, as an escape strategy, when grasped by a predator.

For the adzuki bean beetle (*Callosobruchus chinensis*), Ohno and Miyatake (2007) argue that flying away and entering a state of TI are two alternative tactics that are used in response to predators. In their study, lines of beetles were artificially bred for long or short duration of TI. Ohno and Miyatake measured flying ability in terms of how strongly an experimentally-dropped beetle influenced its downward trajectory (i.e. how far it landed from directly below its release point) and found that those bred for long-duration TI had weaker flying ability, while the lines bred for greater flight ability showed correlated lower TI duration. The exact mechanism underlying this genetic correlation was not determined, but the authors speculated that there may be competition for resources between investment in flight muscles and in reproduction; highly-reproductive individuals may thus have poor flying ability and separately (for reasons that are unclear to us) this may relate to a finding that larger individuals show longer duration of TI. Alternatively, Ohno and Miyatake suggest that individuals that show a low propensity for TI may be more active and, thus, more likely to find a means of escape from their rearing facility. As a final suggestion, and their most plausible speculation to our minds, they suggest that TI might be indirectly selected, despite a direct cost, because it is genetically correlated with other traits that confer higher fitness. This is reminiscent of a study by Nakayama and Miyatake (2009), who found that longevity, emergence rate and egg size were all correlated with the extent of TI in the adzuki bean beetle.

Genetic factors relating to the mechanism of TI have also been shown to influence when TI will be deployed as an anti-predatory strategy over fleeing. Miyatake et al. (2008a) found that locomotion and tonic immobility are pleiotropically correlated with a genetic factor related to a biogenic amine in the red flour beetle. Walking distance was significantly lower in strains artificially selected for longer (L-strains) than shorter TI duration (S-strains), and cross-breeding experiments suggested that tonic immobility and locomotor activity have the same genetic basis (Miyatake et al. 2008a). The authors suggest that the alternative behaviours of fleeing or tonic immobility are associated with the pleiotropic effects of a neuroactive substance in this species.

In some cases, aspects of the environment will also impact the adaptive significance of TI relative to other anti-predator strategies. For example, Miyatake et al. (2008b) found that TI occurred more strongly in two species of seed beetle (*Callosobruchus macalatus* and C. *chinesis*) when individuals were kept at lower temperatures. Miyatake et al. interpreted this as a consequence of higher temperatures allowing beetles to use fleeing as an alternative anti-predator behaviour more readily. Similarly, Saxena (1957) found that woodlice (*Armadillidium vulgare*) showed stronger TI at lower temperatures, again presumably a condition where the alternative anti-predator tactic of fleeing was less available. Saxena also showed, though, that woodlice showed stronger TI when lighting was reduced; in this condition, vulnerability to visual predators when stationary was reduced and so remaining immobile was probably a more viable anti-predator strategy.

Further, there is evidence that TI is used flexibly according to how ecological circumstances influence its costs and benefits relative to alternative anti-predator behaviours (most obviously fleeing to a refuge). Arduino and Gould (1984) demonstrated this well in their experiments on domestic chicks. Chicks were exposed to some stimulus—either representing a poor, fair, or good opportunity to escape—for 15 s, followed by 15 s of being manually restrained while still being exposed to the stimulus. This induced TI, the chick was then released and the duration of TI was measured. Arduino and Gould found, in numerous paired comparisons, that TI lasted longer in situations where it was thought that the chick would consider the chance of escape to be lower (e.g. when the stimulus was a hawk model facing the chick), compared to cases when this chance might be considered to be higher (e.g. when the hawk model was facing away from the chick). An earlier study by Gallup et al. (1971) also found that, in young chickens, individuals immobilised by manual restraint in the presence of a hawk model exhibited longer durations of TI than chickens immobilised without the presence of the model. Santos et al. (2010) suggest that *Liolaemus occipitalis* lizards also evaluate predation risk in adopting anti-predator strategies as, in their study of TI experimentally induced by manual handling, TI lasted longer if the human ‘threat’ remained in close vicinity; if a predator remains close, it is less likely that fleeing would be a useful tactic.

As well as likelihood of escape, proximity to a place of safety can also influence whether animals choose to adopt a strategy of TI or attempt to flee from a predator. Ewell et al. (1981) demonstrated in a laboratory that rabbits (*Oryctolagus cuniculus*) decreased the duration of TI as proximity to a human decreased or as proximity to their home cage increased. Likewise, Hennig et al. (1976) found that TI was of shorter duration in Carolina anole lizards (*Anolis carolinensis*) that had been immobilised near large bushes than those immobilised in the absence of nearby protective cover. Further, O’Brien and Dunlap (1975) reported that blue crabs (*Callinectes sapidus*) immobilised on a sandy substrate (in which they could seek cover by burying themselves) exhibited shorter durations of TI than individuals similarly immobilised on a hard surface (on which burying themselves for protection was not possible).

As an important aspect of an individual’s environment, the local predatory community has also been found to serve as a key influence on the evolution and deployment of particular adaptive anti-predatory behaviours such as TI. The predatory community present can influence the anti-predator strategy deployed by prey, with the prey typically carrying out the behaviour most likely to protect it in a predator-prey interaction. Gyssels and Stoks (2005) found that TI in larvae of the damselfly *Ischnura elegans* was more frequently induced experimentally in those from a pond in which fish predators were present compared to those from a pond where this important predatory group was absent. As well as demonstrating a genetic correlation in the laboratory between flight ability and duration of TI discussed previously, Ohno and Miyatake (2007) also found such a negative correlation across 21 wild populations of the adzuki bean beetle. They speculate that one reason for this may be that the two alternate tactics have differential effectiveness against different predators and that the predator communities differ between populations. Additionally, or alternatively, they speculate that one tactic (perhaps flying away) may be both more effective and more costly, and populations that face lower predation pressure are selected to make more use of the cheaper but less effective tactic (TI).

As well as cost-benefit trade-offs surrounding alternative anti-predator behaviours, TI traits may impact the evolution of other important fitness traits. In some species, duration and frequency of TI have been found to negatively correlate with predation experienced, but also negatively correlate with mating success. Nakayama and Miyatake (2010a) bred lines of the red flour beetle *T. casteraneum* for either longer duration and higher frequency TI (labelled the L-strain) or shorter and less frequent (labelled the S-strain). They then demonstrated that males of the L-strain suffered less from predation in an experimental arena with an Adanson’s house jumping spider, but had reduced mating success in a predator-free environment. This latter effect was interpreted by the authors as individuals of the L-strain being, overall, less active and thus encountering females less frequently; a genetic trade-off clearly exists between an individual’s ability to avoid attack and find mates.

Nakayama and Miyatake (2010b) similarly bred L- and S-lines of the adzuki bean beetle and found that the L-strain had reduced activity that translated into reduced mating success in males but not females. This is probably because male mating success is more dependent on frequency of encounters and, thus, on activity levels. Activity levels might also explain the finding (Nakayama and Miyatake 2009) that L-strain individuals had higher longevity, rates of emergence, egg size and faster development. These authors suggest that reduced activity may conserve energy, increasing longevity, and allowing diversion of energy to larger eggs that take less time to develop. So, while longer and more frequent TI may reduce the risk of predation at the cost of reduced mobility and mating opportunities, the resulting energy conservation may ultimately increase individual fitness and offspring fitness.

In investigating the red flour beetle *T. castaneum*, Kiyotake et al. (2014) inferred some different potential costs to TI behaviours. They demonstrated that L-type beetles—selected to show higher frequencies and longer durations of TI—were significantly more sensitive to environmental stressors, such as mechanical vibration and high or low temperatures, than S-type beetles – selected to exhibit lower frequencies and shorter durations of TI. This could have evolutionary consequences for the species, with the prevalence of TI behaviour potentially being linked with exposure to stressors in wild populations. Some of the authors of this study had been involved in a previous work (Miyatake et al. 2004) demonstrating that the frequency of predation by Adanson’s house jumping spider was significantly lower amongst L-type beetles than S-type beetles. However, they also found that the wild population showed a majority of S-type individuals. This suggests that longer and more frequent TI behaviours may bring an advantage in reducing predation but that, in the wild population, being more robust to environmental stressors and changes may be an even greater advantage in terms of overall fitness, such that shorter and less frequent TI behaviours are prevalent.

As another good example of a fitness trade-off relating to TI, Kuriwada et al. (2011) found that mated sweet potato weevil females showed longer duration TI than virgin individuals. The authors interpreted this in terms of a shifting trade-off between avoiding predation and being active to search for mates. They also found that older females decreased investment in TI, which they again interpreted as reduced relative costs to predation in older individuals. Males, however, showed no change with age. Kuriwada et al. argued that there should be a strong effect of increasing age (and, thus, mortality risk from factors other than predation) in females that must not only mate, but also collect food for investment in eggs and find oviposition sites; versus males, who need only find mates. Previously, Kuriwada et al. (2009a) had found an effect of copulation in both males and females. The authors suggest that the difference between studies was that the earlier study tested TI 5 h after the treatment (copulation or control) was imposed, increasing to 30 h in the second study. The authors speculate that the long interval during which no females could be encountered may have caused perception of mating opportunity to decline in males of both treatments, making a treatment effect difficult to pick up. Miyatake (2001a) had previously found that duration of TI in the sweet potato weevil decreased with increased duration of starvation, and that this effect was stronger in males. This last result can be explained by males being more susceptible to mortality through starvation than females. Miyatake (2001b) also observed a sex-difference in TI in the sweet potato weevil. Both sexes showed a reduction in TI at night, which Miyatake suggests might be because most of their predators are visual and so predation risk is generally lower at night. The effect was particularly strong in males, and the author interpreted this as a consequence of copulations occurring at night and males having more to gain from remaining active to obtain multiple matings than females.

In their study of adzuki bean beetles, Hozumi and Miyatake (2005) found that smaller beetles showed shorter duration TI, something the authors interpreted in terms of the shorter natural lifespan of smaller individuals. As touched upon previously, Nakayama and Miyatake (2009) bred this species for either long or short duration of TI. Fecundity was the same in the two lines, but those with longer-duration TI produced bigger eggs, that developed more quickly, were more likely to hatch and produced longer-lived offspring. This was interpreted as TI requiring less energetic investment than fleeing, and the energy saved being diverted into reproduction. However, Nakayama and Miyatake note that their stocks experienced an abundance of food and potential mates. In other ecological circumstances, the energetic savings of TI may have to be traded off against the lost time that could be devoted to finding food and mating.

Fascinatingly, particular aspects of an individual’s environment and personal experiences have also been shown to influence TI variation and propensity. An interesting vertebrate study by Odén et al. (2005) shows that social environment can affect expression of TI. Odén et al. reported that female laying hens showed shorter duration TI in response to standardised human handling if they had been kept in groups with males. The authors interpreted this as males acting as guards and lowering fear levels in the females.

It may also be that experience of previous environmental conditions can influence TI, as well as the currently experienced condition. Tojo (1991) found that common cutworm (*Spodoptera litera*) raised in crowded conditions in their 4th and 5th instar showed reduced periods of TI in their 6th instar compared to those raised in isolation. This might be seen as an adaptive response to a trade-off between avoiding predation and competing for food resources; crowding may favour individuals that succeed in accessing the limited per capita resources available ahead of those that show extended anti-predator responses but limit their mobility. Tojo also reported variation between natural populations in duration of TI and demonstrated, in cross-breeding experiments, that this had a genetic component. In general, variation and plasticity in TI could be driven by evolution since aspects of this behaviour are heritable; recently, for example, Mignon-Grasteau et al. (2017) have shown that some TI traits (i.e., duration and number of inductions) show moderate heritability in broiler chickens (*Gallus gallus domesticus*).

In a study investigating the effects of human contact and intra-specific social learning on TI in guinea pigs (*Cavia porcellu*), de Lima Rocha et al. (2017) found that, firstly, experience of handling and habituation did not prevent TI responses in subjects. However, habituation increased the latency of TI, while handling or habituation decreased the duration of the TI behaviour. Additionally, the cohabitation of unhabituated and habituated animals was found to reduce TI duration in the unhabituated. The authors concluded that experience of human interactions can reduce experimenter fear in guinea pigs and that unhabituated guinea pigs may learn not to fear experimenters by cohabitating with habituated guinea pigs. Experience and social environment can clearly be important in determining the occurrence of and variation in TI behaviours.

Further, the complexity of how TI, as an anti-predatory behaviour, interacts with other parts of an individual’s responses to the environment is demonstrated by the study of Kuriwada et al. (2009a) on the effect of copulations in the sweet potato weevil. In both sexes, encounters with individuals of the opposite sex where copulation was experimentally prohibited did not affect duration of any subsequent TI. However, an encounter leading to successful insemination led to reduced TI length in females but not males. Regardless of whether copulation lead to insemination, females reduced TI after a single copulation, and males after multiple copulations. Kuriwada et al. interpret the behaviour of males as follows. Sex recognition ability is poor in this species and so copulation rather than simply encounters may be needed to suggest to males that there is high current availability of females. Therefore, activity to find females should be prioritised over avoiding predation for a male, since it may take them longer to find and successfully mate with females. For females, copulations may signal high male activity and thus high risk of sexual harassment. Having allowed successful sperm transfer once, a female’s interest in the attentions of further males decreases and, thus, such females will be willing to accept higher predation risk in order to remain active to avoid unwanted male attentions. This interpretation seems plausible, but further work could rule out alternative explanations (e.g. involving inseminated females increasing the priority that they give to feeding relative to avoiding predation). No matter what, this work stands as an example of how TI can be used flexibly dependending on the state of the organism and of how it can be integrated into a complex of other behavioural and life-history traits.

Interestingly, a different study by Kuriwada et al. (2009b) found that mass-rearing over 71 generations in the sweet potato weevil (*Cylas formicarius*) caused no change in the duration of TI. This consistency in TI traits is surprising, since there was no predation pressure during these 71 generations, but the authors speculate that the behaviour was perhaps maintained—despite the lack of predation pressure—simply because TI is not very costly. TI here did not occur at the expense of resources required for other behaviours as the mass-reared weevils were provided with abundant food and had a high chance of successfully encountering mating partners. Additionally, we would add that TI may have been triggered very infrequently in any individuals during these 71 generations, and that there may be little expressed cost in retaining the capacity to perform TI in situations where that capacity is seldom triggered. However, the constancy of TI duration found by Kuriwada et al. (2009b) might not actually be very informative considering how different the captive situation it created was from natural conditions. Kuriwada et al. (2009b) also posit that TI behaviour may have been indirectly selected via positive genetic correlation with other traits that confer higher fitness, as appeared to be the case in Nakayama and Miyatake (2009). We find it likely that TI was not simply maintained due to being an inexpensive trait, but rather due to the influence it appears to have on other fitness factors such as breeding potential, egg size, growth rate, and fecundity.

We have discussed here the many influences TI traits and expression can have on a prey individual’s anti-predator strategies, life history, and overall fitness. The predator’s side of TI is more fully explored in the next section.

## Coevolutionary considerations

There is increasing evidence that TI does have a functional basis in reducing the probability of an attack by a predator ending in the prey’s death. We now turn to consider why TI causes the predator to reduce its attacking, and so how TI may have evolved alongside predation by certain types of predator. Our suggestion is that the TI tactic might be effective against predators that commonly face a situation where they can subdue more than one prey item in quick succession. In this circumstance, TI works by inducing the predator to switch prematurely from one prey item to the next. The trade-off between ensuring a kill and gaining as much energy as possible may make it maladaptive for the predator to invest time in making absolutely sure that the first item is dead when there are multiple other prey items temporarily available in the hunting session. We know of no study that has explored this hypothesis. In order to explore this, we suggest that it would be useful for a systematic study of anecdotal accounts of the observation of TI to explore the percentage of occasions when the predator had the chance to attack another prey item soon after releasing the individual exhibiting TI. Our prediction would be that this percentage will be high.

Additionally, or alternatively, it would be worth exploring whether individuals of species exhibiting TI occur commonly in groups. Evidence that TI can be more effective when predators face multiple prey comes from Miyatake et al.’s (2009) study of the red flour beetle (*T. castaneum*) attacked by Adanson’s house jumping spider in laboratory experiments. Miyatake et al. used two strains, bred for long (L-strain) and short (S-strain) duration of TI, and found that when an L-strain individual was presented to a spider at the same time as a S-strain individual, its likelihood of survival was over four times greater than that when presented individually. When presented in pairs of an L-strain and S-strain individual, the S-strain individual was killed every time, and when presented in a group of five from each strain predation rates were 79% and 21% for S-strain and L-strain individuals, respectively. Additionally, when presented in a pair alongside an individual of another species that does not show TI, 28% of L-strain and 62% of S-strain individuals were killed. From these results, it is clear that exhibiting longer duration TI conferred the greatest survival advantage to L-strain individuals when presented alongside individuals that do not maintain TI for as long. Where a predator faces multiple prey options, there is a preference for mobile prey exhibiting shorter TI duration; therefore, species that deploy TI as an anti-predatory strategy are more likely to experience a survival advantage over other prey when their predator faces multiple prey. Miyatake et al. also reported that levels of potential chemical aversants were not strongly different between the two strains, so the differences in predation rates were probably to do with TI traits and not to a correlated chemical defence. TI can still be effective for single prey individuals, if the predator concerned has evolved to release prey quickly, because it often encounters prey in groups, against which quick release can aid in making multiple kills.

It may also be that TI is ineffective against predators that specialise on a given prey type and have, thus, been able to coevolve effective responses to TI. As an example of this, Gregory et al. (2007) demonstrated TI in the grass snake (*Natrix natrix*) and hypothesised that this would only be an effective tactic against generalist predators that rarely encounter it. From this, Gregory et al. then suggested that grass snakes should less readily show TI in those parts of its range shared by the snake specialist short-toed eagle (*Circaetus gallicus*).

## Thanatosis in other contexts

There are a handful of reports where TI has been described as occurring in contexts other than prey using it as a defence against predators. In fact, the reverse has been observed in two species of predatory cichlid fish—*Haplochromis livingstoni* and *Parachromis friedrichsthalii—*that appear to adopt TI as part of their hunting strategy (McKaye 1981; Tobler 2005). These fish adopt a pose that appears subjectively to mimic a corpse falling through the water column and then lie inert on the substrate. The cichlid “corpse” then appears to attract other fish, that are interpreted as motivated to scavenge from it; such fish are then attacked by the cichlid that “comes to life” when a would-be scavenger swims close to their mouth (McKaye 1981; Tobler 2005). In both studies reporting this predatory tactic, TI was only shown by adults, which McKaye speculates may be because juvenile fish of these species would be sufficiently small to be vulnerable to predation themselves if they lay immobile. A similar tactic involving apparently feigning illness or dying to draw potential prey closer has been reported in the shallow-water predatory comb grouper fish (*Mycteroperca acutirostris*) (Gibran 2004). Although the authors of these studies link the observed behaviours to TI, we see them as being more akin to mimicry and masquerade.

Even more curious, arguably, is the apparent use of TI in the context of reproductive behaviours. Hansen et al. (2008) demonstrated that males of the nursery web spider (*Pisaura mirabilis*) that entered a state of TI during mating were more successful in gaining copulations and gained longer copulations than those males that did not. Sexual cannibalism is rare in this species and, when it does happen, it occurs before the part of the mating sequence when TI is manifest; it is therefore unlikely that this risk is the main driver of males adopting an immobile state. In the nursery web spider, mating is initiated by males offering the female a nuptial gift. While the female consumes this gift, the male begins sperm transfer. The female can, however, interrupt copulation, after which she can either return to eating or depart with the gift (ending the copulation). In Hansen et al.’s study, some males responded to such female interruption by entering a state of TI, which was associated with higher probability of the female returning to feeding, after which the male left its TI state and returned to copulating. This can happen several times during a copulation session. Hansen et al. speculate that there must also be a cost to TI to explain variation in its expression between males. They speculate that this cost might be heightened vulnerability to attack by females, energetic cost of maintaining the pose, or risk of injury if they are dragged across the substrate in this pose by a fleeing female. However, as yet there has been no investigation of these alternatives.

Several other studies have also reported the use of TI in situations related to reproduction. Male mantids of the species *Mantis religiosa* freeze after mating to avoid post-copulatory cannibalism by the female (Lawrence 1992), and robber fly females of the species *Efferia varipes* behave similarly to avoid harassment by males (Dennis and Lavigne 1976). More recently, Shreeve et al. (2006) describe “possum behaviour” in their observations of European Lepidoptera. Following extreme persistence of males in some tribes, non-receptive female butterflies closed their wings, entering a state of TI, and released themselves from the underlying substrate in order to escape male attention. Similarly, Khelifa (2017) has reported apparent sexual death feigning behaviour in female *Aeshna juncea* dragonflies, seemingly acting to avoid male harassment. Khelifa (2017) posits that females entering a TI state in this way may undergo less coercion and therefore survive longer and produce more offspring. Shreeve et al. (2006) suggest that the occurrence of TI behaviours in these contexts could be related to: female mating frequency, male mate-locating mechanisms and the physical structure of habitats where attempted mating occurs. Certainly, both the ecological factors that may influence TI and the trade-offs involved in contexts other than anti-predatory defence warrant greater exploration.

Finally, TI has also been reported as functioning to avoid worker aggression in social insects, rather than just aggression from natural predators seeking a meal. van Veen et al. (1999) discuss the use of TI by gynes in the stingless bee *Melipona beecheii*. In this species, surplus queens are continually produced but are subject to aggression from the workers and usually killed. van Veen et al. noted that TI appeared effective in stopping instances of worker aggression, although it was also noted that no gynes which showed this behaviour went on to successfully be accepted by the colony. The evolution of TI in this context would be interesting to explore, given that it did not contribute to reproductive success. Undoubtedly there will also be other contexts and other taxa in which TI occurs which are yet to be reported.

## Future challenges and opportunities for research

As discussed in this review, TI is clearly a fascinating anti-predatory strategy of which, while we have a few good studies shining light on the topic, there undoubtedly remain a host of unresolved issues and future opportunities for research. A substantial challenge remains to identify and then understand the taxonomic prevalence of this behaviour. Exploring the occurrence of TI in different taxa and what factors influence propensity of exhibiting TI in these different taxa (King and Leaich 2006) is probably a bountiful opportunity for behavioural ecologists. We suspect that TI may often be observed in the field by researchers conducting studies of other aspects of animal biology, and that these incidents go unreported. We would see great value in raising awareness of TI amongst biologists generally and providing some easy means for observations to be logged and collated. We suggest that a valuable way forward in this would be for collaboration between those scientists who have an interest in TI to reach a consensus on such an endeavour. This consensus might involve agreement on (i) a simple definition that would allow non-specialist biologists to identify incidents of TI; (ii) the aspects of a TI manifestation that should ideally be recorded; (iii) design and maintenance of a single website where such data could be inputted; and (iv) vigorous and sustained effort to publicise the initiative widely amongst animal biologists. Such a consensus might usefully agree on how the data from research efforts could best be analysed.

Based on the arguments presented in the section 6 ‘Coevolutionary considerations’, we consider that it would also be of great value to record information on the predators that induce the TI as well as the prey that displays it. This would allow testing of predictions about which predators are more likely than others to induce TI and expand our understanding of the contexts in which the behaviour is likely to occur. However, both prey and predator traits should influence the prevalence of TI, and we would expect (for example) that the behaviour is more common in aggregated prey that have some capacity to flee upon discovery by a predator which can only capture those prey one at a time.

One important issue for TI research, currently, is the dearth of experiments in the wild. The last 20 years have seen a huge leap in our understanding of TI (at least in insects) driven by some very effective laboratory studies. However, in interpreting such studies we must remember that captive animals experience a very unnatural (lack of) predation regime. They have also often experienced nothing but this artificial world for their whole life and, indeed, may often be bred from stock that have been removed from natural predation pressures for generations. While a study by Passos et al. (2017) recently showed that differences in TI behaviour in populations of wild and captive populations of golden mantella frogs (*Mantella aurantiaca*) were principally due to body condition rather than rearing environment, the authors rightly argue that this result does not mean that the influence of unnatural rearing environments can be dismissed in terms of their influence on behaviours. Further, in most studies TI is induced by humans rather than a natural predator. There is a strong need for us to find systems in which we can study TI in the wild. Ever-improving technology involving tracking animals and on-board cameras should be powerful weapons in such an endeavour. Head-mounted cameras that allow predators of the size of a domestic cat to hunt for prey unimpeded are now very cheaply and widely available. Laboratory studies have also highlighted behavioural, physiological and life-history correlates of TI, and we would benefit now from finding a suitable model system in which to explore whether similar relationships can be found under naturally-occurring conditions.

Of course, there is still an important place for laboratory study, and here - for example - we would encourage an increased focus on how TI influences predators’ subsequent behaviours. It might be, again, that there is a place for advanced technology here. An attraction of remote-controlled robotic simulated prey is that the form and extent of TI can be manipulated while controlling all potentially confounding variables, in a way that is very difficult in experiments with living prey. In fact, in the EU it is illegal to conduct experiments involving predation of vertebrate prey, and so there is no alternative except to utilise technology in such a way for vertebrate studies.

As an additional development to the topic, the occurrence of TI in humans is a further, more recent avenue of research, with opportunities for the fields of psychology and potentially important implications for post-trauma therapy treatments (Marx et al. 2008; Volchan et al. 2011, 2017). Immobility reactions of the human defensive cascade seem to have parallels with those proposed for non-human species (Volchan et al. 2017), and through developing understanding of the physiological mechanisms of the phenomenon in humans (Volchan et al. 2011) it is possible that scientists could go on to alleviate symptoms in victims of traumatic stress.

## Conclusions

1. We define tonic immobility (TI) as the unlearned adoption of a motionless posture by a prey individual triggered by physical contact or very close proximity of—not injury inflicted by—a predator (or other antagonist). The posture does not reduce the sensory ability of the predator to locate or identify the prey, or reduce the physical vulnerability of the prey if the attack is pursued. The state of motor inhibition is maintained for a time even after release by the predator, and when in this state the prey exhibits reduced responsiveness to external stimulation (although monitoring of the environment can still occur). In the absence of mortality or injury during TI, the prey can recover its original physiological state on emerging from TI.
2. TI has been described in a wide range of taxa, but there remain relatively few quantitative accounts of the phenomenon. It has likely been, and could in the future be, observed in many more taxa than are currently reported.
3. A great range of different physiological mechanisms can be involved in controlling TI, both in vertebrates and invertebrates, and there are many possible ways in which the resulting behaviour may confer protection from predators.
4. There is strong evidence that for prey species utilising TI as a defence against predators, but we suspect there are far more examples to be found in the wild.
5. Evolutionary and ecological trade-offs affect both the prevalence of and variation in TI behaviours. Many factors; including the mobility requirements, reproductive opportunities, genetic correlations, perceived predation risk, and the physical and social environment; may influence whether TI is the most advantageous behaviour to adopt in any given situation.
6. If TI has a functional basis in reducing the probability of an attack by a predator ending in the prey’s death, the coevolution of TI with certain predators is likely to influence its occurrence. We suggest that TI might be effective against predators that commonly face a situation where they can subdue more than one prey item in quick succession.
7. In a few, rare reports, TI has also been described as occurring as part of reproductive behaviours and avoiding aggression in various taxa. It may well have other intriguing functions that are yet to be reported.
8. A challenge for the study of TI remains to identify and then understand the taxonomic prevalence of this behaviour. There is also a need to develop understanding of the contexts in which TI occurs and increase the scientific literature describing wild studies, perhaps with the help of new technologies.
